# Condylar Aplasia and Hypoplasia: A Rare Case

**DOI:** 10.1155/2013/745602

**Published:** 2013-03-24

**Authors:** Peeyush Shivhare, Lata Shankarnarayan, Usha, Mahesh Kumar, Malliger Basavaraju Sowbhagya

**Affiliations:** Department of Oral Medicine and Radiology, Raja Rajeswari Dental College, and Hospital, Rajiv Gandhi University, Ramohalli Cross, kumbalgodu, Bangalore, Karnataka 560074, India

## Abstract

Aplasia of condyle is very rare, when this condition not seen as a part of a syndrome. We report a case of condylar aplasia on the right side and hypoplasia on the left side in a 21-year-old female. The patient reported to the department with a chief complaint of underdeveloped lower jaw. Clinical examination, conventional radiographs, and 3D CBCT images revealed complete absence of condyle on the right side and hypoplasia on the left side.

## 1. Introduction

The temporomandibular joint (TMJ) is one of the most complex joints of the human body. It is considered a ginglymus diarthrodial joint capable of both rotational and translatory movements. It consists of the mandibular condyle and the articular eminence of the temporal bone. The condyle is very special because the expression of mandibular growth is provided by mandibular condyle. In compared to other diarthrodial joints, during prenatal life the TMJ lags morphologically behind other synovial joints in both the timing of its appearance and its progress, so that at birth the joint is still largely underdeveloped. The TMJ first appears in the 8th week of gestation, when two separate areas of mesenchymal blastemas appear near the eventual location of the mandibular condyle and glenoid fossa [1, 2]. Bone and cartilage are first seen in the mandibular condyle at approximately the 10th gestational week. First condylar blastema developed from which the mandibular condyle cartilage, the aponeurosis of the lateral pterygoid muscle, and the disc and capsule component composing the lower portion of the joint are derived. Next is the temporal blastema, which eventually forms the articular surface of the temporal component and the structures of the upper portion of the joint. The mandibular condyle and temporal blastemas begin their growth at relatively distant sites; they then move towards each other as the joint develops by the 12th week. At birth, the articular surfaces of both the mandibular condyle and temporal bones are covered with fibrous connective tissue. Later, this tissue is slowly converted to fibrocartilage as the fossa deepens and the mandibular condyle develops under functional influences [3, 4].

Growth disturbances in the development of mandibular condyle may occur in utero late in the first trimester and may result in disorders such as aplasia or hypoplasia of the mandibular condyle. As compared to hypoplasia, hyperplasia of the mandibular condyle is not visible at birth and seems to be gradually acquired during growth [5].

## 2. Case Report

A 21-yr-old female was presented to the oral medicine and radiology department with a chief complaint of underdeveloped lower jaw, which was first noticed during childhood and gradually progressed. Due to unfavorable socioeconomic conditions, it was not possible to get the treatment done for the patient. At the anamnesis there was no history of any trauma or any systemic diseases. Patient's parents gave a history of consanguineous marriage. There was no family history of the present problem. 

**Figure 1:**
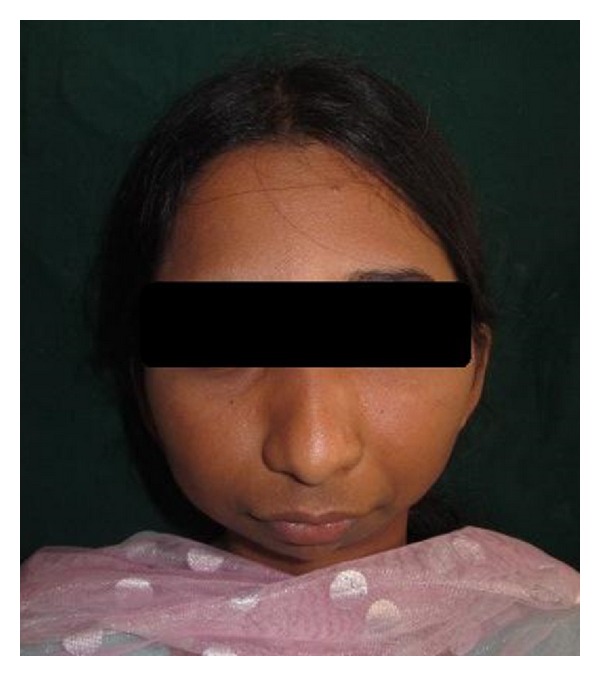
Frontal view of patient.

**Figure 2:**
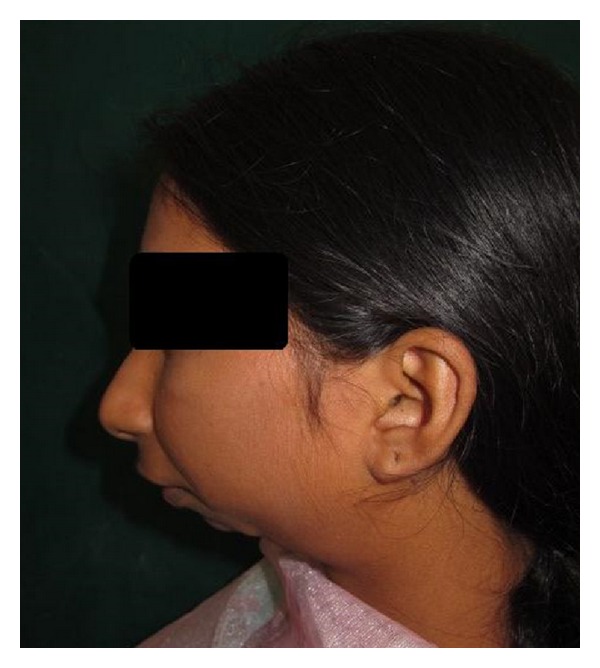
Lateral view of patient shows severe retruded mandible.

**Figure 3:**
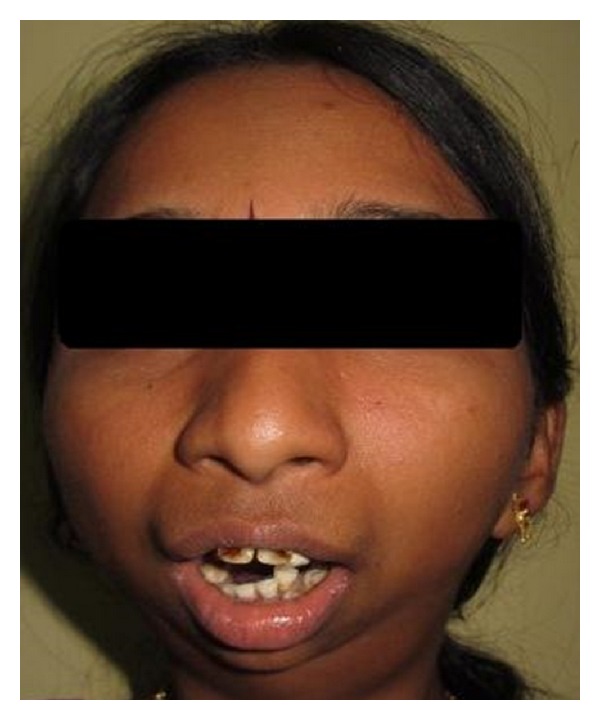
Restricted mouth opening.

**Figure 4:**
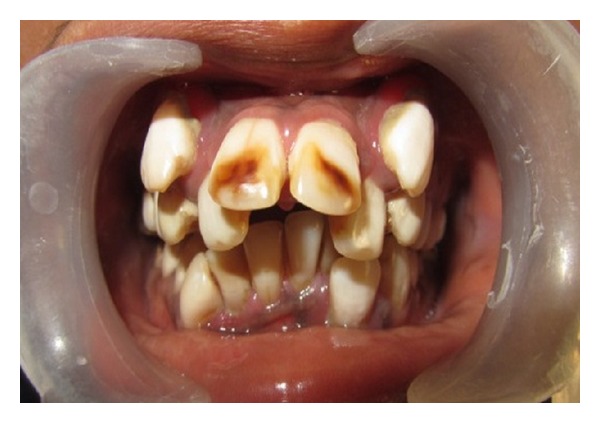
Severe crowding in upper and lower anteriors with fluorosis.

General Physical examination did not reveal any abnormalities. Her vital signs were within normal limits. Extraoral examination revealed facial asymmetry with severe retruded mandible giving a bird face appearance (Figures 1 and 2). Mouth opening was restricted (10 mm) with no deviation or deflection (Figure 3). On palpation condyles were not detected on both sides. Intraorally there was crowding in upper and lower anterior teeth with increased overjet and overbite (Figure 4). Molar relation was Angle's Class II bilaterally. She had deep palate and generalized enamel hypoplasia (fluorosis). No other important clinical extraoral or intraoral findings were observed. Based on clinical findings, a provisional diagnosis of bilateral ankylosis and differential diagnosis of bilateral condylar hypoplasia or aplasia were given. 

**Figure 5:**
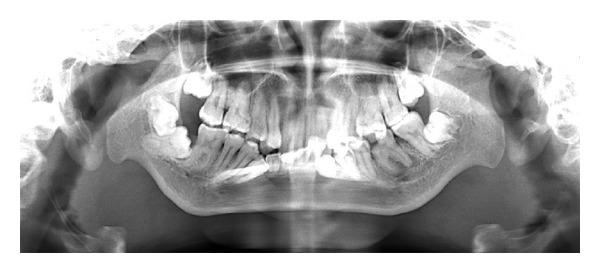
OPG shows condylar aplasia on the right side, condylar hypoplasia on the left side, prominent antigonial notch, and hypoplasia of mandible.

**Figure 6:**
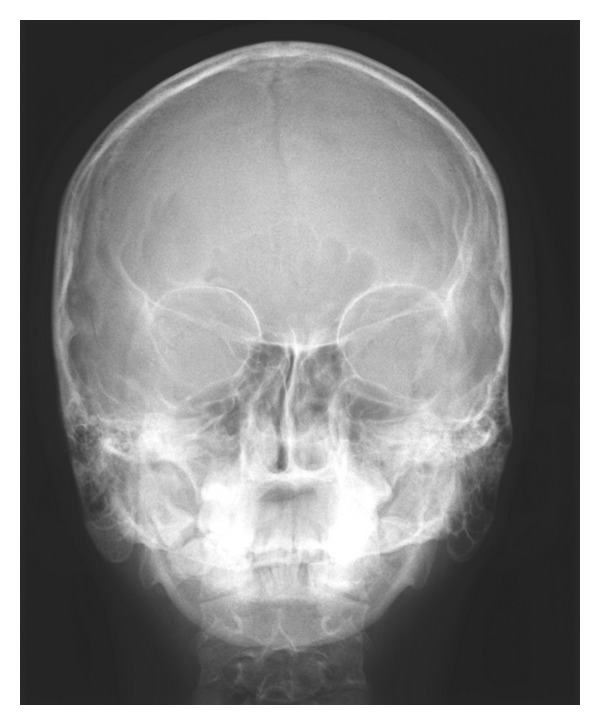
PA view reveals no significant asymmetry.

**Figure 7:**
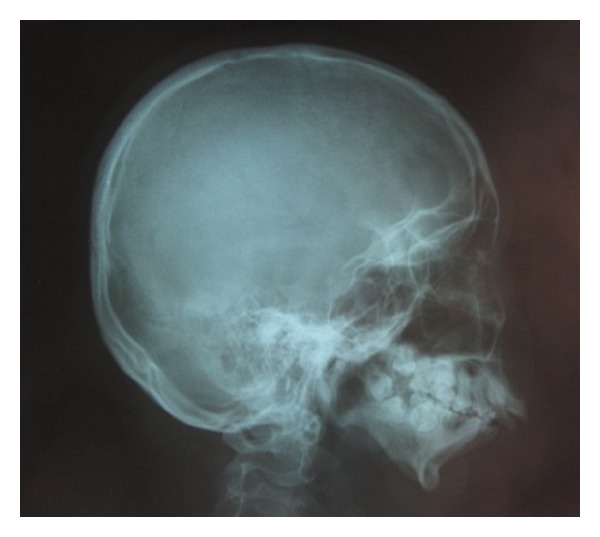
Lateral skull. Radiograph shows severe retruded mandible.

**Figure 8:**
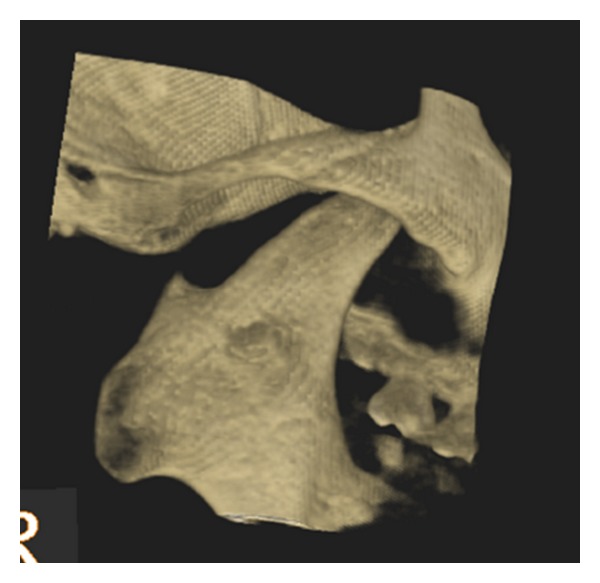
3D CBCT right oblique lateral shows absence of glenoid fossa and complete absence of the condyle.

**Figure 9:**
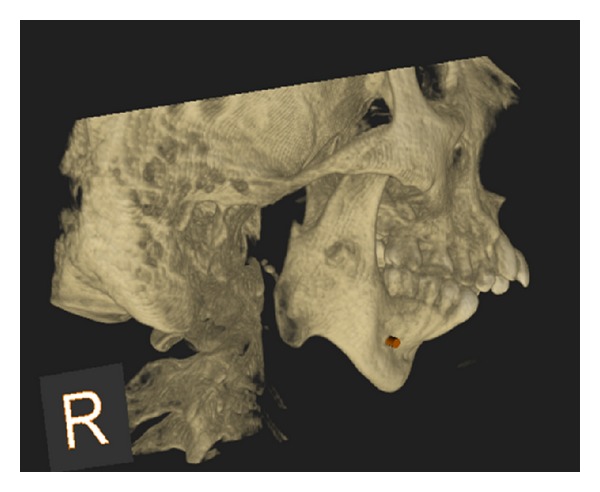
3D CBCT right lateral shows absence of glenoid fossa and complete absence of the condyle.

**Figure 10:**
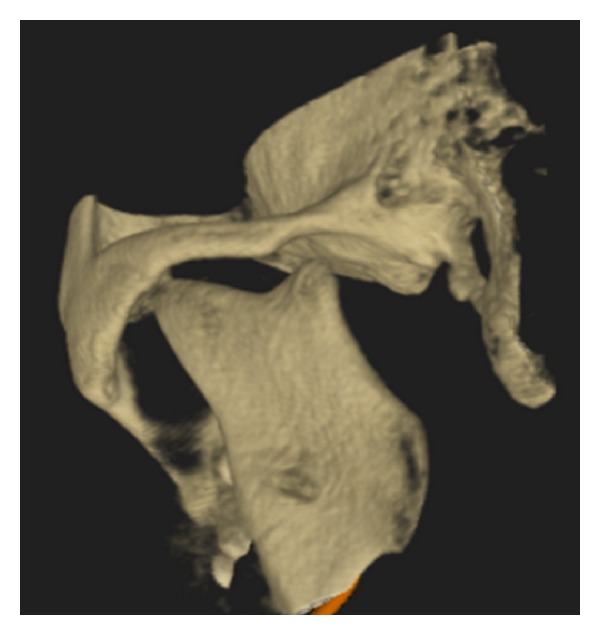
3D CBCT left oblique lateral—shows hypoplasia of glenoid fossa and condyle.

**Figure 11:**
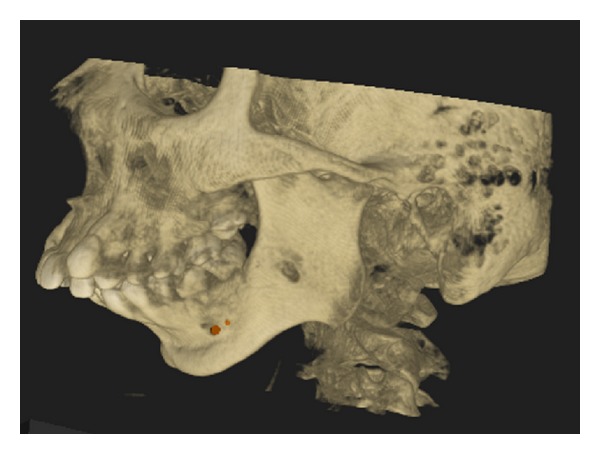
3D CBCT left lateral—shows hypoplasia of glenoid fossa and condyle.

After clinical examination, radiographic examinations were performed. Panoramic radiograph showed complete absence of condyle on the right side and rudimentary condyle on the left side. Glenoid fossa was not developed on the right side and underdeveloped on the left side. Antegonial notch was prominent bilaterally (Figure 5). PA view findings were inconclusive (Figure 6). Lateral skull views showed severe retruded and micrognathic mandible (Figure 7). Findings of panoramic radiograph were confirmed by lateral skull radiographs. CBCT was advised for additional information. CBCT also confirmed the findings of OPG and lateral skull radiographs (Figures 8, 9, 10, and 11). After radiographic confirmation patient was advised complete systemic evaluation and referred to general medicine, cardiology, ophthalmology, ENT, and orthopedics to rule out any syndromes. Medical evaluation revealed no abnormalities. Based on the clinical and radiographic findings, a final diagnosis of nonsyndromic agenesis of the right condyle and hypoplasia of the left condyle was given. Patient was referred to oral surgeon and orthodontist for the best possible treatment.

## 3. Discussion 

The congenital deformities and developmental abnormalities of the mandibular condyle can be classified as hypoplasia or aplasia, hyperplasia, and bifidity. Hypoplasia or aplasia of the mandibular condyle indicates underdevelopment or nondevelopment associated mainly with various craniofacial abnormalities. These may be either congenital or acquired [5].

Congenital (primary) condylar hypoplasia is characterized by unilateral or bilateral underdevelopment of the mandibular condyle and usually occurs as a part of some systemic condition originating in the first and second branchial arches, such as Mandibulofacial dysostosis (Treacher Collins syndrome), Hemifacial microsomia (first and second branchial arch syndrome), Oculoauriculovertebral syndrome (Goldenhar syndrome), Oculomandibulodyscephaly (Hallermann-Streiff syndrome), Hurler's syndrome, Proteus syndrome, Morquio syndrome and Auriculocondylar syndrome [5–8].

As a rule, in each of these conditions some soft tissue manifestations accompany the condylar agenesis and/or condylar malformations [9].

Acquired (secondary) condylar hypoplasia takes place if the condyle is injured during active growth, because of which development may be arrested. The most common causes are mechanical injury, such as trauma (before the age of 2), infection of the joint itself or the middle ear, childhood rheumatoid arthritis, radiotherapy, and parathyroid hormone-related protein deficiency which affect bone formation and chondrocyte differentiation [5, 9, 10].

Several authors confirmed that mandibular deficiency can occur without any defined etiology [11]. Aplasia of the mandibular condyle without any other facial malformations is an extremely rare condition [8].

The cases of nonsyndromic mandibular condyle aplasia have been previously reported by Krogstad [9], Prowler and Glossman [11], Akihiko et al. [12], Santos et al. [13], Bowden Jr. and Kohn [14], Canger and Celenk [15] and so forth. Our case also presented condylar aplasia and hypoplasia without any other features suggestive of any syndrome.

 The TMJ develops from initially widely separated temporal and condylar blastemata which appear at about the 8th week of conception. Eventually they grow towards each other and ossify to form a functional joint by about the 20th week of intrauterine life [5]. In our case, total absence of the condyle and glenoid fossa on the right side and hypoplastic condyle and glenoid fossa on the left side constitute an evidence that the defect originated in the prenatal period.

Various treatment approaches have been proposed for treating condylar aplasia and possibilities for influencing mandibular growth. Most of the time it is treated by multimode with the help of oral surgeon, general surgeon, plastic surgeon, and orthodontist [9, 15, 16].

The treatment could then be a costochondral graft transplant, preferably before the growth spurt, orthognathic surgery at the end of the growth period, or both [16]. Krogstad reported that effective results were obtained through the application of a form of orthodontic activator which aimed to swing the mandible to the unaffected side and promote formation of a mandibular condyle, albeit irregular in shape [9]. Surgery is often required, but the timing and regimen of this choice is still an issue to be resolved [15]. 

## 4. Conclusion 

In conclusion we report a rare case of total condylar aplasia on the right side and condylar hypoplasia on the left side, not related to any clear pathological disorder. This case of unknown etiology was thoroughly examined; based on clinical and radiographic findings, we suggest that this case is of congenital origin. Nonsyndromic condylar hypoplasia and aplasia are exceedingly rare conditions and very few case reports are published till date. In this context, our case is an important addition to the literature. Early detection and prompt treatment are imperative to restore esthetics and thus provide psychologic benefit to these patients.
